# How have unintended pregnancies and contraceptive use among adolescent girls and young women changed in Uganda? Evidence from the 2014 and 2019 PMA national surveys

**DOI:** 10.1371/journal.pone.0321235

**Published:** 2025-04-29

**Authors:** Simon P.S. Kibira, Mary Nakafeero, Mathew Amollo, Ronald Ssenyonga, Rawlance Ndejjo, Phil Anglewicz, Melody Kukundakwe, Mabel Luzze, Samuel Kagongwe, Victor Guma, Vivian Zalwango, Fredrick Edward Makumbi

**Affiliations:** 1 Department of Community Health and Behavioral Sciences, School of Public Health, Makerere University, Kampala, Uganda; 2 Department of Epidemiology and Biostatistics, School of Public Health, Makerere University, Kampala, Uganda; 3 Department of Social Work and Social Administration, College of Humanities and Social Sciences, Makerere University, Kampala, Uganda; 4 Department of Public Health, Faculty of Health Sciences, Muni University, Arua, Uganda; 5 Department of Disease Control and Environmental Health, School of Public Health, Makerere University, Kampala, Uganda; 6 Department of Population, Family and Reproductive Health, Johns Hopkins Bloomberg School of Public Health, Baltimore, Maryland, United States of America; PLOS: Public Library of Science, UNITED STATES OF AMERICA

## Abstract

Unintended pregnancies among adolescent girls and young women (AGYW), and any pregnancy among adolescent girls are still a challenge, especially in Sub-Saharan Africa and Uganda. We assess prevalence of unintended pregnancy in Uganda, associated factors and contraceptive use following unintended pregnancy among adolescent girls and young women in 2014 and 2019 in Uganda. Data are from the 2014 and 2019 performance monitoring for action (PMA) surveys. There were 1,479 AGYW reporting ever/ or current pregnancy in the two surveys, 780 in 2014 and 699 in 2019. Data included socio-demographics and pregnancy intendedness. Descriptive analyses were conducted stratified by adolescent girls and young women and compared between surveys. The percent of unintended pregnancies was determined as the number of AGYW reporting unintended pregnancy divided by eligible participants. A weighted comparison of the prevalence of unintended pregnancies was made between the surveys, and statistical significance determined at a 5% type-1 error rate. All analyses were conducted with Stata version15 using svy surveyset methodology accounting for complex survey design. Relative to 2014, the 2019 survey showed a significant reduction in percent of AGYW reporting ever pregnant or given birth, 60% to 49%, p = 0.007; a decline in unintended pregnancy among adolescent girls, 52% to 42%, p = 0.049, and among young women with secondary education, 36% to 13%, p = 0.001. Conversely, the 2019 survey showed significant increase in contraception among those ever pregnant, 26% to 40%, p < 0.001; higher in young women (30% to 47%, p = 0.001) compared to adolescent girls (16% to 25%, p = 0.005). The commonest contraceptive methods were short-acting at both surveys, while the long-acting methods significantly increased among young women (20% to 35%, p = 0.003). The decline in unintended pregnancies was consistent with increased use of contraceptive methods. Although we observed a significant decline in unintended pregnancy among adolescent girls, the proportion reporting unintended pregnancy remains high.

## Introduction

Although there is increased equity in access to contraception and use [1], nearly half of pregnancies among all women of reproductive age globally, estimated at 121 million each year are still unintended. [2]. This crisis is more pronounced in less developed countries, particularly in sub-Saharan Africa [3–5], with disparities further between countries in the region [4]. In countries such as Kenya and Ethiopia, a quarter of women of reproductive age in past and recent studies reported their last pregnancy as unintended [4,6,7]. Disparities also exist in rates within countries by age, marital status, residence and socioeconomic status [4]. The rates are much higher in Uganda where 46% of women who had a child in the 5 years preceding a 2022 national survey reported the (last) pregnancy as unintended [8].

Adolescent girls in Uganda report even higher rates of unintended pregnancies than women in other age groups. While the unintended pregnancy rate reported in the 2022 survey was 46%, among women of reproductive age in Uganda, for adolescent girls 15–19 years old, it was 63% [8]. Such unacceptably high rates of unintended pregnancies highlight the need for interventions to save the future of this young generation. It should be noted that, among adolescent girls, pregnancies of any kind, intended or unintended, are undesirable given their associated risks [9] and consequences [10]. They are also particularly costly both to families and the countries. In Uganda it is estimated that these pregnancies cost the country $365m in the year 2020 alone[11]. Yet, adolescent girls’ fertility rates in Sub-Saharan Africa remain more than twice the global average. For example, in 2019, 5.8 million adolescent girls age 15–19 years gave birth in the region [10]. In Uganda specifically - the focus of this paper - the teenage pregnancy rates have persistently averaged 25% for more than two decades [12,13].

Adolescent girls are likely to engage in unprotected casual pre-marital sexual intercourse as a result of lack of choices [10], or being taken advantage of through defilement [6,9,14,15] and may have limited access to effective contraceptive information and methods [16,17]. For unintended pregnancies among adolescent girls and young women (AGYW), several factors have been associated; early sexual debut (before age 18 years), being out of school, lower education levels, limited awareness about contraception, and previous sexual experiences [4,18–20]. The high rates of adolescent girls pregnancies are a key development matter affecting personal and National progress [10,11]. Many unintended pregnancies among AGYW are likely to end in unsafe abortions [21–24] and the resultant challenges. It has also been associated with experiencing depressive symptoms [25], dropping out of school for female learners, high unwanted fertility, malnutrition for children born in resource limited households, and increased risk for maternal and child mortality [4,9,18].

One of the most effective ways to prevent unintended pregnancies among sexually active AGYW, is ensuring access to contraceptives that offers great benefits [26]. Yet, although there are advances, contraceptive use remains sub optimal among all women in Uganda, [8,12]. In Uganda, a country with one of the youngest populations (44% below age 15 years) [27], harnessing of a demographic dividend [28] is one of the developments matters that the National Planning Authority, Uganda government ministries and implementing partners are prioritizing. It should be noted that Uganda has a short window of opportunity to harness a demographic dividend, whose attainment depends on improving indicators in several areas including health [29] and particularly reproductive health [30]. Ensuring a substantial reduction in both unintended pregnancies among young women and any pregnancy among adolescent girls is key to improve women and children’s health and wellbeing, which will eventually impact on development [30]. Without scientific evidence of changes over time, it is impossible to ascertain progress is made, and which groups are left behind. It is therefore important to monitor these changes, to inform the demographic dividend roadmap [31] interventions.

The purpose of this paper is to estimate the changes in unintended pregnancy in Uganda among AGYW age 15–24 years and the associated factors using the 2014 and 2019 national surveys. We also set out to determine the contraceptive use status of AGYW reporting a pregnancy and the choice of contraceptive methods following an unintended pregnancy. We believe that this study provides the much-needed scientific evidence to show progress of the family planning and population programs on addressing the unintended pregnancies among AGYW and identify those still left behind who may need more targeted or strengthened interventions.

## Methods

### Design and sample

**Fig 1:**
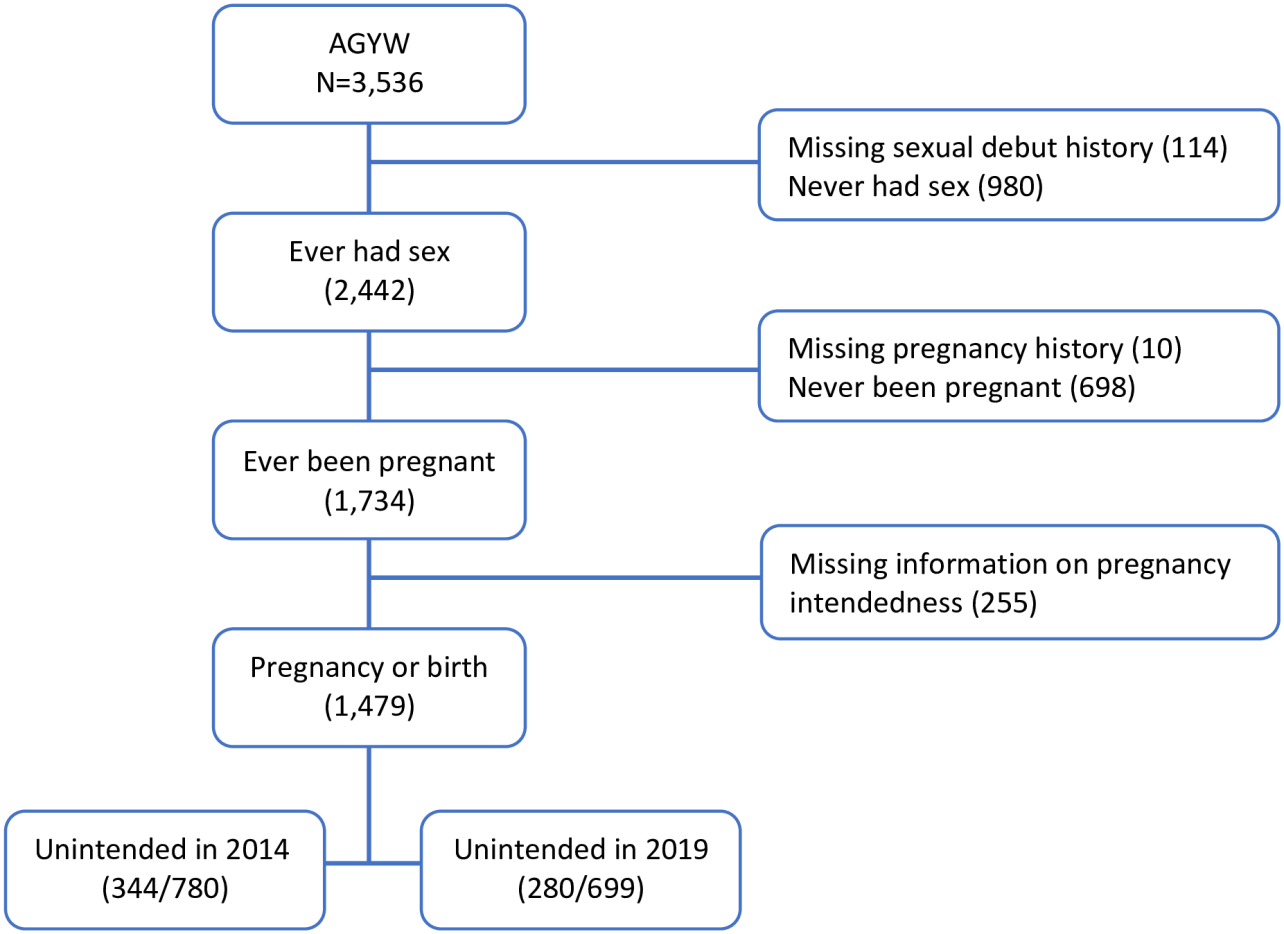
Flow diagram for the sample analyzed.

We analyzed data from two rounds (2014 and 2019) of the Performance Monitoring and Accountability (currently known as Performance monitoring for Action (PMA)) project national surveys using a sub sample of AGYW aged 15–24 years. PMA nationally representative household surveys in Uganda have been implemented annually since 2014. The 2014 and 2019 surveys included 4,802 and 4,928 households, 3,782 and 4,586 women aged 15–49 years who completed interviews in these households, respectively. This paper only focused on 1,479 AGYW age 15–24 years who had ever been pregnant (780 in 2014 and 699 in 2019) and had complete responses to the variable of intendedness of the most recent or current pregnancy. (Fig 1). The two survey years were chosen because they provided a 5-year window that allowed the complete aging of the adolescent girls from 2014 into young women category in 2019.

Both PMA2014 and PMA2019 surveys were conducted in 110 enumeration areas, each with approximately 200 households, selected probability proportional to size of the 10 statistical regions of Uganda, at the time. Multi-stage sampling was employed in both national surveys where in the first stage enumeration areas were drawn by the Uganda Bureau of Statistics from a 2014 National housing and population census master frame in both survey years. In the 2014 survey, in each enumeration area, listing and mapping was conducted by study staff, and 44 households were randomly selected in the second sampling stage. Household characteristics including members were recorded, and all eligible females, age 15–49 years, were contacted and consented for interviews by female enumerators. For the 2019 survey, the study staff returned to the same set of 110 enumeration areas as those that were selected in a previous round (in 2018). Each household structure that was originally selected for the 2018 survey was contacted and enumerated. The same procedures in the household applied for all eligible females 15–149 years. Data were collected from 10^th^ May to 27^th^ June for the year 2014 survey, and from 13^th^ May to 11^th^ July for the year 2019 survey.

The detailed methodology for the PMA surveys in Uganda and other countries is further described in more detail elsewhere [32,33].

### Statistical analysis

The primary outcome was unintended pregnancy when an AGYW reported that she did not want to have any (more) children at the time of the last birth or if she did not want to conceive at the time of the most recent or current pregnancy. Prevalence of unintended pregnancies was computed as the number of AGYW reporting an unintended pregnancy divided by total number of AGYW that reported ever been pregnant. Contraceptive use status variable included whether a modern, or any family planning method was used by AGYW who had ever delivered and were not currently pregnant, as well as for women who reported being sexually active. Two variables were created: reporting history of contraceptive use and current use of contraception. Contraceptive use methods were categorized as; traditional (rhythm method, withdrawal, and any other traditional method), modern short acting (injectables, oral contraceptive pills, emergency contraception, male and female condoms, standard days/ cycle beads, and lactation amenorrhea methods (LAM)) and modern long-acting methods (implants and intra uterine devices (IUD)). For purposes of this analysis, the 2014 and 2019 surveys, which are 5 years apart, are considered. Changes in adolescents’ (15–19 years) outcomes are compared between these two surveys. Similarly, the young women’s (20–24 years) outcomes are compared between these two surveys. The adolescents (15–19 years) in the 2014 survey are independent of those in 2019, so are the young women (20–24 years) between the two periods, because the surveys were 5 years apart.

All percentages were weighted in the survey years. We calculated weights for the analysis given this was complex design survey. Primary sampling units (enumeration areas) and their corresponding selection probabilities were provided by Uganda Bureau of Statistics. Following the mapping and listing in each of the selected 110 enumeration areas, and selection of the fixed number of 44 households per enumeration area, the selection probabilities of households were obtained. Following the interviews within each household, the probability of interviewing each eligible respondent was also obtained. The product of these probabilities was used to obtain the final sampling probability attributed to each respondent. The inverse of the final sampling probability was used to obtain the analysis weights.

Age-stratified analysis was performed to compare the percentages of unintended pregnancies between 2014 and 2019. Change was defined as the difference in percentage of the outcome and the corresponding 95% confidence interval of the difference was provided. A two-sample test of proportions was used to determine statistical significance at 5% level of significance.

### Regression analysis for factors associated with unintended pregnancies

We implemented mixed effects modified Poisson regression analysis with random intercepts, an unstructured covariance structure and fixed effects covariates to determine prevalence ratios (PR) as a measure of association for the identification of factors associated with unintended pregnancies. The high prevalence of unintended pregnancies necessitated the use of modified Poisson regression. Appropriateness of multilevel modelling was investigated by obtaining the intra cluster correlation coefficient (ICC). An ICC value of 0.02 was obtained indicating low correlation among observations in the same EA. Although the ICC was low, for individual women within clusters (enumeration areas), we continued with multilevel modelling. Even with small ICC values, in this multistage sampling design at a national level, multilevel modelling can still be meaningfully implemented to minimize clustering effects that may bias results if not accounted for.

Four models were fitted in the mixed effects regression in assessing the factors associated with unintended pregnancies among AGYW: model 1 (the null model), without any independent variables; model 2, with individual level variables (age, age at first sex, marital status, highest education level); model 3, individual level variables plus wealth status, a household level variable and; model 4, variables in model 3 plus residence, and enumeration area level variable. Interaction between the year of the survey and residence was investigated in the final Model 5. Collinearity among the independent variables was checked and determined if it did or did not exist, by obtaining the variance inflation factors (VIF); none of the variables had a VIF greater than the recommended value of five [34]. Adjusted prevalence ratios are presented with their corresponding 95% confidence intervals. Stata version 15 was used for the analyses.

### Ethical considerations

The surveys were approved by the Makerere University School of Public Research and Ethics committee (protocol 081) and the Uganda National Council for Science and Technology (SS3400). Written informed consent was obtained from all the participants. For adolescent girls aged 15–17 years, written assent was obtained after parental/guardian consent. However, for the adolescent girls of this age who are pregnant or have given birth are recognized as emancipated minors, and therefore do provide consent in such surveys.

## Results

### Characteristics of adolescent girls and young women

In 2014, 60% (213) of the adolescent girls 15–19 years reported to have ever been pregnant or given birth, and 49% (200) in 2019. For the young women (age 20–24years), 82% had been pregnant in 2014, while 80% in 2019.

Table 1 shows the demographic characteristics of AGYW (15–24 years) who reported to have ever been pregnant or given birth at the time of the two surveys, 2014 (780) and 2019 (548). In both surveys, most AGYW were in union, had no formal education or only attained primary level, had 1^st^ sex when at least aged 15 years, and lived in rural areas. There was a slight variation in wealth quintiles, with majority of the adolescent girls reporting a pregnancy or birth, living in the lowest quintile households in 2014, and middle quintile in 2019, while majority of young women lived in the highest quintile households.

**Table 1:** Demographic characteristics of women age 15-24 who had ever been pregnant in Uganda, 2014 and 2019 surveys.

	Adolescent girls, 15–19		Young women, 20–24	
	2014	2019	2014	2019
	Weighted Freq (Col%)	Weighted Freq (Col%)	Weighted Freq (Col%)	Weighted Freq (Col%)
**Status of last birth/current pregnancy**				
Intended	105 (47.8)	118 (58.2)	320 (57.3)	300 (60.6)
Unintended	115 (52.2)	85 (41.8)	239 (42.7)	195 (39.4)
**Age at first sex**				
Less than 15	57 (26.0)	25 (12.4)	80 (14.4)	52 (10.6)
15 and above	163 (74.0)	177 (87.6)	479 (85.6)	442 (89.4)
**Marital status**				
Married	156 (70.8)	142 (70.2)	463 (82.7)	412 (83.4)
Not married	64 (29.2)	60 (29.8)	97 (17.3)	82 (16.6)
**Highest level of education attained**				
None or Primary	176 (79.8)	201 (99.4)	387 (69.2)	462 (93.5)
Secondary or Tertiary	45 (20.2)	1 (0.6)	172 (30.8)	32 (6.5)
**Residence**				
Urban	30 (13.7)	26 (12.7)	132 (23.7)	110 (22.2)
Rural	190 (86.3)	177 (87.3)	427 (76.3)	385 (77.8)
**Wealth quintile**				
Lowest quintile	58 (26.3)	52 (25.6)	92 (16.5)	127 (25.6)
Lower quintile	51 (23.1)	47 (23.2)	94 (16.9)	91 (18.4)
Middle quintile	41 (18.6)	48 (23.6)	121 (21.6)	92 (18.6)
Higher quintile	38 (17.0)	39 (19.3)	117 (20.9)	91 (18.5)
Highest quintile	33 (15.0)	17 (8.3)	135 (24.2)	93 (18.8)
**Total (%)**	**100**	**100**	**100**	**100**
**N** *(weighted and normalised)*	221	203	559	494

There was some variation in characteristics of AGYW who reported ever giving birth or been pregnant, by survey year and age categories. Just over a quarter of adolescent girls reported sex before age 15 years (26%) in 2014 and 12% in 2019, while among young women, it was 14% in 2014 and 11% in 2019. Variations by education level indicate one in five adolescent girls had secondary or higher level in 2014 (20.2%) and less than one percent in 2019 (0.6%), while among young people in 2014, 31% had secondary level and only 7% in 2019. There were no substantial variations in residence within the age groups across the survey years.

### Difference in unintended pregnancy in 2014 and 2019

Table 2 shows the difference observed in the unintended pregnancy among AGYW between 2014 and 2019 national surveys in Uganda.

**Table 2:** Prevalence and change in the prevalence of unintended pregnancy among women 15-24 years between 2014 and 2019 PMA surveys in Uganda.

	Adolescent girls, 15–19				Young women, 20–24			
	2014%	2019%	Change (95% CI)	p-value	2014%	2019%	Change (95% CI)	p-value
Overall	52.2	41.8	−10.4 (−20.9,−0.03)	**0.049**	42.7	39.4	−3.3 (−10.1,3.5)	0.336
***Age at first sex***								
Less than 15	56.6	25.3	−31.3 (−53.3,−9.6)	**0.005**	49.7	44.6	−5.1 (−24.0,13.8)	0.597
15 and above	50.7	44.1	−6.6 (−18.3,5.2)	0.271	41.5	38.8	−2.7 (−10.0,4.5)	0.453
***Marital status***								
Married	43.6	28.7	−14.9 (−26.8,−2.9)	**0.014**	41.3	36.2	−5.1 (−12.4,2.3)	0.178
Not married	73.1	72.6	−0.5 (−18.5,17.3)	0.949	49.4	55.2	5.8 (−10.3,21.9)	0.479
***Highest education level attained***								
None or Primary	54.2	42	−12.2 (−23.3,−1.1)	**0.032**	45.5	41.2	−4.3 (−11.9,3.2)	0.260
Secondary or Tertiary	44.4	0			36.3	13.2	−23.1 (−37.4,−8.9)	**0.001**
***Residence***								
Urban	57.9	22.5	−35.4 (−59.3,−11.5)	**0.004**	42.2	21	−21.2 (−33.3,−9.0)	**0.001**
Rural	51.3	44.6	−6.7 (−18.1,4.6)	0.243	42.8	44.6	1.8 (−6.0,9.5)	0.658
***Wealth quintile***								
Lowest quintile	63	49.4	−13.6 (−33.6,6.3)	0.180	55.2	45.7	−9.5 (−23.8,4.7)	0.189
Lower quintile	54.1	35.3	−18.8 (−39.9,2.3)	0.081	51.7	45.6	−6.1 (−22.1,9.7)	0.448
Middle quintile	53.7	47.4	−6.3 (−28.9,16.2)	0.582	32.6	42.5	9.9 (−4.5,21.4)	0.178
Higher quintile	40.9	38.2	−2.7 (−26.5,21.2)	0.826	42.9	36.8	−6.1 (−21.6,9.5)	0.447
Highest quintile	41.3	28.4	−12.9 (−44.5,18.9)	0.425	36.8	24.2	−12.6 (−27.0,1.9)	0.088

Overall, there was a reduction in AGYW reporting their last birth or pregnancy as unintended in 2019 compared to 2014 survey. Among adolescent girls, 52% in 2014 and 42% in 2019 reported their last birth or pregnancy was unintended. This represents a 10-point significant reduction in this percent (p = 0.049). Among young women, 43% in 2014 and 39% in 2019 reported their last birth or pregnancy was unintended, representing a 3.3-point reduction, although this was not statistically significant (p = 0.336).

For AGYW in all wealth quintiles, there were non-statistically significant reductions in unintended pregnancies across all wealth quintiles. This was similarly observed among young women, except for those in the middle quintile households that had an 9.9-point increase, although not statistically significant as well.

There was a 5.8-point increase in the percent of young women not in union, reporting unintended pregnancies from 2014 (49%) to 2019 (55%), although this was not significant.

### Contraceptive use status in 2014 and 2019 survey years and the type of methods following unintended pregnancy

Table 3 shows the percent AGYW who have ever given birth that report ever use of contraception and, those currently using a method of contraception. It also shows AGYW who report sex in the 12 months preceding the surveys (sexually active) who were using a method of contraception in the two surveys, as well as the method types.

**Table 3:** Contraceptive use status following unintended pregnancy among adolescent girls and young women, age 15-24 years in Uganda, 2014 and 2019 surveys.

	Adolescent girls (15–19)			Young women (20–24)		
	2014	2019		2014	2019	
	% (95% CI)	% (95% CI)	p-value	% (95% CI)	% (95% CI)	p-value
**Ever used contraception among those ever pregnant or delivered**						
Yes	26.1 (21.4,31.3)	46.4 (40.9,51.9)	<0.001	48.7 (44.6,52.7)	70.6 (66.8,74.4)	<0.001
**Currently using contraceptive method among those ever pregnant or delivered**						
Yes, any method	15.6 (11.6,20.6)	25.4 (20.5,30.9)	0.005	30.3 (26.5,34.6)	46.9 (42.6,51.3)	<0.001
Yes, modern	14.8 (10.9,19.8)	21.9 (17.5,27.1)	0.034	29.9 (25.9,34.1)	40.9 (36.7,45.3)	<0.001
**Currently using contraception among sexually active, not pregnant**						
Yes, any method	20.2 (14.2,27.9)	35.1 (27.4,43.7)	0.006	38.0 (32.8,43.5)	55.2 (49.9,60.0)	<0.001
Yes, modern	18.7 (12.9,26.3)	31.5 (24.2,39.9)	0.015	37.3 (32.1,42.8)	47.8 (24.6,53.1)	0.006
**Method type used among those sexually active, not pregnant**						
Traditional	7.4 (1.8,25.8)	10.2 (3.4,26.5)	0.709	3.1 (1.1,8.6)	13.4 (9.0,19.4)	0.001
Short acting	76.1 (56.9,88.5)	63.6 (48.6,76.3)	0.251	77.4 (68.8,84.2)	51.9 (44.6,59.0)	<0.001
Long acting	16.5 (6.8,34.9)	26.2 (15.9,40.1)	0.300	19.5 (13.2,27.8)	34.8 (28.1,42.0)	0.003

There was an increase in the percent of AGYW reporting ever use of contraception across the two survey years. Only 26% of adolescent girls reported ever use of contraception in 2014, while in 2019, 46% reported ever use. Among young women, the percent reporting ever use rose from 49% in 2014 to 71% in 2019.

The percent reporting current use among those who had ever been pregnant or delivered was also substantially higher in both the adolescent girls and the young women in 2019 compared to 2014. Adolescent girls reporting current use of any method in this category were 16% in 2014 and 25% in 2019, while among young women, it was 30% and 47% in 2014 and 2019, respectively.

The percent of AGYW who were sexually active (sex in the last 12 months) reporting current use of any method was also substantially higher in 2019 compared to 2014; from 20% to 35% among adolescent girls, and 38% to 55% among young women for any method.

The type of methods most used by the sexually active AGYW, at the time of the survey, were short acting modern methods in both 2014 and 2019 surveys. However, the percent reporting long-acting methods increased in both groups; from 16% to 26% among adolescent girls, and 20–35% among young women, in 2014 and 2019 respectively.

### Factors associated with unintended pregnancies among adolescent girls and young women

We found a significant reduction in unintended pregnancies between 2014 and 2019, aPR = 0.45 (95% CI: 0.24–0.81, p-value = 0.008) after adjusting for age, marital status, education level, wealth, residence, and age at first sex (Table 4). Prevalence of unintended pregnancies was significantly lower among AGYW with a secondary or tertiary education, aPR = 0.78 (95% CI: 0.62–0.99, p-value 0.042) compared to those with no education or a with a primary education. It was also lower among AGYW living in wealthier households when compared to those from the poorest households. Prevalence of unintended pregnancies was significantly higher among the unmarried AGYW, aPR = 1.59 (95% CI: 1.42–1.79, p-value<0.001) as well as among rural dwellers in the 2019 survey year aPR = 2.05 (95% CI: 1.10–3.82). Results shown in Table 4.

**Table 4:** Regression analysis for factors associated with unintended pregnancies among adolescent girls and young women, age 15-24 years in Uganda, 2014 and 2019 surveys.

Characteristic	Crude	Adjusted	p-value
	PR (95% CI)	PR (95% CI)	
***Age***			
15–19	1.00	1.00	
20–24	0.88 (0.76–1.02)	0.98 (0.84–1.14)	0.768
***Age at first sex***			
Less than 15	1.00	1.00	
15 and above	0.92 (0.76–1.10)	0.97 (0.79–1.18)	0.783
***Marital status***			
Married	1.00	1.00	
Not married	1.55 (1.37–1.74)	1.59 (1.42–1.79)	<0.001
***Highest education level attained***			
None or Primary	1.00	1.00	
Secondary or Tertiary	0.77 (0.63–0.95)	0.78 (0.62–0.99)	0.042
***Survey year***			
2014	1.00	1.00	
2019	0.89 (0.74–1.07)	0.45 (0.24–0.81)	0.008
***Residence***			
Urban	1.00	1.00	
Rural	1.29 (0.98–1.69)	0.87 (0.66–1.14)	0.311
***Interaction between survey year and residence***			
2019*Rural		2.05 (1.10–3.82)	0.023
***Wealth quintile***			
Lowest quintile	1.00	1.00	
Lower quintile	0.95 (0.80–1.13)	0.95 (0.79–1.12)	0.507
Middle quintile	0.84 (0.67–1.04)	0.79 (0.64–0.98)	0.032
Higher quintile	0.76 (0.61–0.96)	0.76 (0.61–0.95)	0.016
Highest quintile	0.65 (0.49–0.87)	0.71 (0.53–0.96)	0.028

The intraclass correlation coefficient (ICC) shows that the variability in unintended pregnancies between individual and household level models reduced from 4% to 2%. (Table 5) This means that half of the total variability from the individual level was explained by the household level variable (wealth quintile). There was no change in ICC between the household (wealth quintile) and EA (residence) level variables.

**Table 5:** Mixed effects modified Poisson regression analysis for unintended pregnancies among adolescent girls and young women 15-24 years in Uganda, 2014 and 2019 surveys.

	Model 1	Model 2	Model 3	Model 4	Model 5
ICC	0.02	0.04	0.02	0.02	<0.01
**EA variance**	0.03	0.04	0.02	0.02	0.00

## Discussion

This study using nationally representative survey data estimated the changes in unintended pregnancy among AGYW in Uganda between the years 2014 and 2019 and determined the contraceptive use status among those reporting ever giving birth or pregnant at the time of the survey. Overall, there were reductions in the percent of both adolescent girls and young women reporting; a pregnancy or ever have given birth, and an unintended pregnancy between the two survey years. In line with these reductions, we report significant increases in the percent reporting ever use of contraception and those who were using contraception at the time of the surveys. More AGYW were using short acting methods of contraception, although the percentage using long-acting methods increased in 2019 compared to 2014.

There was a substantial reduction in the percent of adolescent girls who reported a pregnancy or ever birth in 2019 compared to 2014 survey, as well as a non-significant reduction among young women 20–24 years. In a country that started its demographic transition just over a decade ago [35], a reduction in teenage births will have a lasting effect on fertility rates. The National Planning Authority in Uganda postulates that, if the fertility declines are accelerated, the age structure will transform with a bulge in the working-age population providing a window of opportunity for rapid economic growth [29]. This assumes the right social and economic policies are developed and corresponding investments are made. Having children later exposes women to fewer years of reproduction and eventually fewer expected births. Uganda has invested in universal secondary education since 2007 [36]. This intervention could be improving reproductive health outcomes, which may include fewer reported pregnancies and adolescent girls’ births, when children stay in school longer than before. Among the young women, the gains were not as high as among adolescent girls. This could mean that the younger cohorts are experiencing accelerated fertility reductions than those before. This is vital for Uganda’s targets to reduce population growth rate to 2.4 by 2040 [37], and harness the demographic dividend [30].

We also observed a significant reduction in adolescent girls reporting an unintended pregnancy in 2019 compared to the 2014 survey. This reduction could imply that the adolescent girls in 2019 had more information about pregnancy prevention than the ones five years earlier. Another possible explanation is the desire for education attainment and better living standards, that could be changing over time, which yield a reduction in overall total fertility rates. The effect of education on contraceptive demand [38], and unintended pregnancy has been profiled before [39]. By residence as an equity dimension, adolescent girls in urban areas had a significantly higher reduction compared to those in rural areas. This could be because the urban areas have more channels for reproductive health information and access to methods and may have better quality of services like education. Negative reproductive health outcomes like higher rates of unintended pregnancies, and teenage births are much higher in rural settings [40,41]. It should be noted that even with increasing urbanization, most adolescent girls still live in rural settings in Uganda, and efforts to rapidly improve outcomes in the context of equity need to focus on them as well.

The direct determinant of unintended pregnancy is, not using a contraceptive method [42]. It is vital to ensure AGYW who are sexually active but do not want to have children (yet), use a method of contraception to realize their reproductive rights. We found a substantial increase (1.7 times for adolescent girls, and 1.4 times for young women) in the percent who have ever given birth, reporting ever use of contraception between 2014 and 2019. An increase in ever use of contraception is important because ever use is predictor for future use [43], among those who are not currently using a method. Adolescent girls and young women who have had a pregnancy or are sexually active should have an early exposure to contraceptive information. Reductions in adolescent girls’ pregnancy rates and unintended pregnancy are more likely to arise from use of contraception than reductions in sexual activity as seen elsewhere [44]. There was also a significant increase in AGYW reporting current use of contraception, among those who had ever been pregnant or delivered, as well as among those who were sexually active. When AGYW recognize the need to minimize undesirable outcomes like unintended pregnancies and seek to delay child birth [41], they are likely to demand for contraceptive services. Our findings on increased contraceptive prevalence may imply this demand. The desire to prevent unintended pregnancy has been reported as a key motivator of contraceptive use among young people in Sub-Saharan Africa [45]. Interventions focusing on AGYW family planning services including information may be in higher demand for the current cohorts, and barriers [16] ought to be eliminated to further harness the gains. When AGYW meet their reproductive choices, the benefits will be more impactful over generations.

Among sexually active AGYW, most used short-acting modern methods in both 2014 and 2019 surveys, but we observed an increase in those reporting use of long-acting methods. One of the barriers to use of contraceptives among AGYW reported before, are provider attitudes [45,46], but these could be improving, with more acceptability. However, the compromise may be to provide short-term methods for adolescent girls, and even young people, as opposed to long-acting methods, because of health worker attitudes or limited capacity [16,17,47] at lower levels where AGYW are more likely to access services. Long-acting methods are documented to have higher efficacy, continuation rates, and satisfaction rates than the short-term methods among adolescent girls opting for them [48], while short-acting methods are more prone to discontinuation [49]. AGYW ought to have choices of any method they want to use, as long they receive complete method information index; told of side effects, what to do when they experience the side effects, told of availability of other methods, and told if they can switch or if the method is permanent. With this comprehensive information, they can choose their most suitable method. The observed increase in use of long-acting methods may be one step towards voluntary method mix in this age group. A variety of methods available to AGYW with uninterrupted supply, will improve contraceptive uptake, facilitate ease to switch and minimize discontinuation. After all, greater availability of a number of contraceptive methods increases contraceptive use over time [50]. With the current expansion towards other venues that open access to family planning self-care-oriented methods, adolescent girls (especially) need not be left behind, given the rising demand for contraceptive methods.

The strength of this paper is that it provided evidence from nationally representative sample surveys of households selected from the same enumeration areas. The results provide a national picture and can guide programming to improve outcomes in reproductive health. This study has some limitations. The two time periods are not a cohort, and therefore the changes over time do not reflect the same group aging from adolescence (15–19) to 20–24years. The adolescent girls in 2014 are therefore not necessarily the same as the young people in 2019. Also, as the PMA surveys sample was not designed to be regionally representative, a comparison between regions was not possible. However, because regions have rural and urban settings, the results about residence (rural/urban) can be utilized to provide insights within regions for settings that meet either classification. Further, future surveys could incorporate qualitative data, especially in a sequential explanatory design, for a deeper understanding of the reasons for adolescent girls’ pregnancies, and the barriers to contraceptive use following pregnancy.

## Conclusion

The results provide hope that the outcomes among the young generations are improving. First the evidence of a reduction of the undesirable outcomes of unintended pregnancies and births among adolescent girls. Second, the increase in contraceptive use among those who have experienced pregnancy or childbirth. Third, the increase in use of long-acting methods following birth will provide more protection against repeat pregnancies. However, the study also unmasks inequities, with unintended pregnancies more pronounced among AGYW in rural areas, those with no education or primary, and living in households of the lowest wealth quintile. These differences in equity dimensions point to the areas that need more focus to leave no AGYW behind.

These findings have policy and program implications. The government and its partners should harness the gains made to ensure progress towards the goals of 2040 and beyond are achieved. This can be through ensuring uninterrupted access - minimizing stock outs - to contraceptive options, including providing information and comprehensive counselling to prevent any pregnancy among adolescent girls and unintended pregnancies among young women, mindful of the inequities evidenced in this paper. For adolescent girls that still get pregnant, strengthening immediate post-partum family planning intervention for them could minimize repeat pregnancies. A focus on the young generation will yield lasting positive outcomes, far beyond reproductive health.
